# Retraction Note: A Zinc Morpholine Complex Prevents HCl/Ethanol-Induced Gastric Ulcers in a Rat Model

**DOI:** 10.1038/s41598-024-73115-2

**Published:** 2024-09-25

**Authors:** Suzy M. Salama, Nura Suleiman Gwaram, Ahmed S. AlRashdi, Shaden A. M. Khalifa, Mahmood A. Abdulla, Hapipah M. Ali, Hesham R. El-Seedi

**Affiliations:** 1 Department of Biomedical Science, Faculty of Medicine, University of Malaya, 50603 Kuala Lumpur, Malaysia; 2 Department of Chemistry, Faculty of Science, University of Malaya, 50603 Kuala Lumpur, Malaysia; 3 Department of Experimental Hematology, Karolinska University Hospital, SE-141 86 Stockholm, Sweden; 4 Department of Molecular Biosciences, The Wenner-Gren Institute, Stockholm University, S-106 91 Stockholm, Sweden; 5 Department of Otolaryngology-Head and Neck Surgery, Kumamoto University, 1-1-1 Honjo, Kumamoto, Japan; 6 Division of Pharmacognosy, Department of Medicinal Chemistry, Uppsala University, Box 574, 75 123 Uppsala, Sweden

Retraction of: *Scientific Reports*10.1038/srep29646, published online 27 July 2016

The Editors have retracted this Article. After publication, concerns were raised regarding the data presented in Figure 11. Specifically,

- Figure 11e appears to overlap with Figure 4d in^1^. These Panels represent tissues taken from animals which underwent different treatments;
- A portion of Figure 11g appears to overlap, when re-scaled, with a portion of Figure 6c in^2^. These Panels represent tissues taken from animals which underwent different treatments;
- A portion of Figure 11c appears to overlap, when re-scaled, with a portion of Figure 4c in^3^. These Panels represent tissues taken from animals which underwent different treatments.

The Authors were unable to provide the original raw data underlying these figures. The Editors therefore no longer have confidence in the results and conclusions reported.

Hesham R. El-Seedi did not explicitly state if they agree or disagree. The Editors were not able to confirm the current contact details of Suzy M. Salama, Nura Suleiman Gwaram, Ahmed S. AlRashdi, Shaden A. M. Khalifa, Mahmood A. Abdulla, and Hapipah M. Ali.

## References

[1] Sidahmed, H. M. A. *et al.* RETRACTED ARTICLE: Gastroprotective effect of desmosdumotin C isolated from Mitrella kentii against ethanol-induced gastric mucosal hemorrhage in rats: possible involvement of glutathione, heat-shock protein-70, sulfhydryl compounds, nitric oxide, and anti-Helicobacter pylori activity. *BMC Complement Altern. Med.*** 13**, 183. 10.1186/1472-6882-13-183 (2013). 23866830 10.1186/1472-6882-13-183 PMC3765280

[2] Nordin, N. et al. Anti-ulcerogenic effect of methanolic extracts from Enicosanthellum pulchrum (King) Heusden against ethanol-induced acute gastric lesion in animal models. *PLoS ONE*** 9**(11), e111925. 10.1371/journal.pone.0111925 (2014). 25379712 10.1371/journal.pone.0111925 PMC4224391

[3] Nazarbahjat, N. et al. Antioxidant properties and gastroprotective effects of 2-(ethylthio)benzohydrazones on ethanol-induced acute gastric mucosal lesions in rats. *PLoS ONE*** 11**(6), e0156022. 10.1371/journal.pone.0156022 (2016). 27272221 10.1371/journal.pone.0156022 PMC4896442

